# IM4Equity: an implementation science meta-framework for community-engaged partnerships to advance health equity

**DOI:** 10.1186/s12913-025-12537-8

**Published:** 2025-03-26

**Authors:** Lilian G. Perez, James L. Merle, Justin D. Smith, Alex R. Dopp, Amy G. Huebschmann

**Affiliations:** 1 Behavioral and Policy Sciences Department, RAND, Santa Monica, CA USA; 2 Department of Population Health Sciences, Division of Health System Innovation and Research, Spencer Fox Eccles School of Medicine, University of Utah, Salt Lake City, UT USA; 3 Division of General Internal Medicine, University of Colorado School of Medicine, University of Colorado Anschutz Medical Campus, Aurora, CO USA; 4 University of Colorado Adult and Child Center for Outcomes Research and Delivery Science (ACCORDS), Aurora, CO USA; 5 Ludeman Family Center for Women’s Health Research, Aurora, CO USA

**Keywords:** Implementation science, Framework, Health equity, User-centered design, Community engagement

## Abstract

**Background:**

Implementation determinant frameworks identify factors that may impede or facilitate implementation of evidence-based innovations (EBI) in new contexts, including diverse community settings. For health equity initiatives, variations in which frameworks are used make synthesis and identification of shared determinants difficult, including equity constructs. Further, such frameworks are rarely informed by community partner input. We describe the development of an equity-centered meta-framework that centers community-engagement called IM4Equity (Crosswalk of 4 IMplementation Frameworks to advance health Equity) as part of the Disparities Elimination through Coordinated Interventions to Prevent and Control Heart and Lung Disease Risk (DECIPHeR) Alliance, comprised of seven research-community partnerships across the U.S.

**Methods:**

We conducted a crosswalk of determinants (domains and constructs within domains) from the Exploration, Preparation, Implementation, and Sustainment framework; Practical, Robust Implementation and Sustainability Model; updated Consolidated Framework for Implementation Research; and Health Equity Implementation Framework. We reviewed original source publications and resources to identify determinants from each framework, which informed a prototype figure. We obtained feedback on the figure with a user-centered design approach with DECIPHeR research teams and community partners, plus framework developers. We used thematic analysis to summarize the feedback and inform iterative development of supporting materials to guide community partner engagement in informing and applying IM4Equity (e.g., blank framework template, guidance for completing the template).

**Results:**

IM4Equity identifies shared and unique domains: intra- and extra-organizational contexts, characteristics of individuals involved in implementation, innovation characteristics, bridging factors, implementation process, and implementation phases. We identified examples of shared constructs for each domain and example factors that may improve health equity or maintain systems of oppression (e.g., structural racism). Feedback sessions identified two areas for improving the usability of IM4Equity, which we addressed in the final figure and supporting materials: 1) design and appropriateness (e.g., language) and 2) approach for integrating community partner perspectives.

**Conclusions:**

IM4Equity highlights key overlapping determinants across existing frameworks, which can promote shared learning across health equity initiatives. IM4Equity is one of the first meta-frameworks to promote co-creation and application of an implementation framework with community partners, which may help inform more equitable implementation measures and strategies to advance health equity.

**Supplementary Information:**

The online version contains supplementary material available at 10.1186/s12913-025-12537-8.

## Background

Advancing health equity in communities that disproportionately experience social disadvantage is one of the greatest challenges of our century [1–4]. The urgency to address inequities in health and healthcare warrants holistic (i.e., addressing multiple barriers while leveraging assets), culturally-appropriate interventions guided by community input to ensure the design, implementation, and evaluation address community priorities [5, 6]. Although such interventions have high potential to advance health equity, they often fall short because of gaps in translating effective interventions, which are often tested in relatively high-resource settings, to more diverse community settings, and inconsistent political will to support their implementation and sustainment. Yet, large-scale implementation of these interventions is necessary for broader and more sustained public health impacts, especially for achieving health equity [7]. However, the translation of “what works” in health research to community settings has historically taken too long for too little uptake [8], and is often disproportionately delayed in reaching communities most impacted by inequities.

Whereas implementation science as a field emphasizes careful consideration of contextual influences on successful translation of evidence-based innovations (EBIs), particularly factors across multiple socio-ecological levels (e.g., environment, policies), recent publications highlight the critical need to better center equity as a key goal of implementation research and practice [2–4]. Several implementation science theories, models and frameworks (hereafter, termed “frameworks” for shorthand) focus on contextual enablers and/or barriers to implementation success, such as characteristics of the EBI and individuals involved as well as local resources and needs [9]. For example, widely used frameworks that consider multilevel determinants include the Consolidated Framework for Implementation Research (CFIR) [10, 11]; Exploration, Preparation, Implementation, and Sustainment (EPIS) framework [12, 13]; and Practical, Robust Implementation and Sustainability Model (PRISM) [14–16]. Understanding multilevel implementation determinants is key to ensure there is strong organizational and external (e.g., community) support for EBI implementation and sustainment. However, few frameworks explicitly consider equity constructs such as the culture and lived experiences of those receiving the EBI (e.g., cultural values, experiences of structural racism and discrimination) [2, 3, 17–20]. Two 2023 scoping reviews found that there are over 140 implementation science frameworks [21] and only 12 have an equity focus [22]. Given creating new frameworks is not desirable, guidance is needed on how to better infuse equity considerations into existing frameworks and to move towards meta-frameworks for parsimony in the field. Such guidance is critical to advance the science of equity-centered implementation research.

Few publications provide guidance on how to integrate equity in implementation frameworks. One landmark example to date is Woodward et al.’s important work developing the Health Equity Implementation Framework (HEIF) [23]. There is framework-specific guidance for HEIF and other frameworks on how to consider equity [17, 18, 24, 25], but we are not aware of frameworks that provide guidance on engaging community partners in the identification of equity-related implementation determinants. Community partner perspectives can inform what equity means from their cultural context and can inform identification of implementation determinants that should be prioritized, measured, and addressed. Thus, to ensure that “successful” implementation is driven by those who will be most impacted by an EBI, guidance is needed on how to center community perspectives (e.g., organizational leaders, residents, patients) and promote collaborative specification of the relevant determinants for a given implementation framework and EBI. This contrasts with the more typical “top-down” approach of traditional implementation exploration and planning efforts that collect data from communities to identify contextual determinants, but rarely engage them in applying determinant frameworks [26–28]. Challenges with the design of most frameworks, such as use of technical jargon and terminology, have made it difficult to engage partners to this degree [28]. Community engagement would open up new opportunities to develop a better-informed understanding of implementation determinants, which can ensure communities share power in deciding what gets evaluated and addressed to improve translation of an EBI and maximize impacts on health equity [25].

Although equity-centered frameworks such as HEIF are filling an important gap to advance health equity by expanding the array of contextual determinants considered, additional limitations exist beyond a lack of community engagement in its creation. First, the constructs in determinant frameworks, including equity-focused ones, need to better reflect the priorities and experiences of communities [28]. Second, many frameworks were developed for use in healthcare settings (such as HEIF); thus, the contextual determinants of other settings with wide reach in underserved communities, such as schools or faith-based organizations, are not clearly operationalized. Third, with the wide range of frameworks to choose from, health equity-focused initiatives often end up using different frameworks, which creates challenges in comparing findings across efforts and limits understanding of common determinants across implementation contexts (e.g., geographic or community settings, intervention delivery settings).

To address the aforementioned gaps, the Disparities Elimination through Coordinated Interventions to Prevent and Control Heart and Lung Disease Risk (DECIPHeR) Alliance [29], comprised of seven research-community partnered Implementation Research Centers, sought to develop a determinant meta-framework [30] that centers equity and community engagement, and could apply or align with projects across a variety of contexts. The result was IM4Equity (Crosswalk of 4 IMplementation Frameworks to advance health Equity). This article aims to describe our process of 1) integrating four implementation determinant frameworks into the IM4Equity meta-framework and 2) eliciting feedback from DECIPHeR research teams to improve the appropriateness and utility of the meta-framework for community-engaged projects addressing health equity. We discuss potential advantages of IM4Equity, adaptations for community settings (clinics, schools, faith-based settings), and additional guidance for supporting community engagement in operationalizing and applying IM4Equity. Further, we discuss ongoing gaps and potential next steps for the field to build capacity for communities to apply determinant frameworks like IM4Equity as part of health equity focused research-community partnerships. IM4Equity may especially be valuable for multi-site initiatives and research consortia where understanding of shared determinants can help inform next steps for addressing the most important drivers of health equity.

## Methods

### Overview of the DECIPHeR Alliance

In September 2020, the DECIPHeR Alliance received funding from the Center for Translation Research and Implementation Science (CTRIS) branch of the National Heart, Lung, and Blood Institute of the U.S. National Institutes of Health [29, 31]. The Alliance is comprised of seven Implementation Research Centers (IRCs) based at universities across the U.S.: 1) the University of California, Los Angeles; 2) the University of Colorado; 3) the University of Illinois at Chicago; 4) Northwestern University; 5) the University of Michigan/Johns Hopkins University; 6) New York University; and 7) Tulane University. Each IRC involves academic-community partnerships seeking to reduce cardiopulmonary inequities by conducting implementation research to improve the delivery of EBIs. The settings and community partners across each IRC vary and include health care systems, community health center organizations, faith-based organizations, and schools. There is also a Research Coordinating Center (RCC) at the University of North Carolina at Chapel Hill. The RCC was engaged in the present work by having members represented in the focus groups as well as the Implementation Science and Community Engagement Subcommittees, who were engaged in providing feedback on IM4Equity. This study was determined as exempt from full review and approved by the RAND Institutional Review Board. Informed consent from all participants was waived.

The DECIPHeR Alliance is a 7-year cooperative agreement using a biphasic funding mechanism. The first phase, UG3 (2020–2023), involved community-engaged exploration of context to inform implementation planning for a hybrid effectiveness-implementation trial. Specific UG3 phase activities included: a) Identifying high burden communities/populations, b) conducting needs assessments, c) establishing community engagement mechanisms (e.g., advisory boards), d) identifying implementation strategies to be tested during the UH3 phase, and e) preparing to implement and deliver selected EBIs during the UH3 phase. The second phase (UH3) is the implementation phase (2023–2027), during which each of the seven IRCs apply findings from the UG3 phase to test implementation strategies for optimally and sustainably delivering multi-level EBIs to reduce or eliminate cardiopulmonary health disparities in tobacco use, uncontrolled hypertension, or pediatric asthma [29]. The DECIPHeR Alliance also includes methodologic subcommittee working groups, such as the Implementation Science Subcommittee and a Community Engagement Subcommittee. During the UG3 phase, the Alliance’s Implementation Science Subcommittee identified a need to work with community partners to assess the contextual determinants of each IRC’s EBI, including those that influence equity such as structural racism and its downstream implications. In addition, given the goal to promote shared learning across the Alliance, we aimed to identify common domains and/or constructs across the frameworks being used by each IRC. These needs inspired our efforts to develop IM4Equity.

### Frameworks included in IM4Equity crosswalk

IM4Equity was developed by integrating four implementation determinants frameworks. Specifically, we selected EPIS, PRISM, and CFIR, which the DECIPHeR IRCs were using to guide their community-engaged planning phase. We also included the HEIF because it offers guidance on determinants related to health equity (e.g., culture and community strengths; biases towards minoritized groups). Table 1 presents a summary of the four frameworks and the sources used to review each.

**Table 1:** Description of individual frameworks integrated in IM4Equity

Framework	Key domains	Sources reviewed
EPIS	• Inner/outer context • Nature of innovation • Role of innovation/practice developers • Phases of implementation process • Bridging factors between inner and outer setting	• Papers describing EPIS, including updates over time [12, 13] • Episframework.com website • Example interview guides asking about EPIS determinants
PRISM	• External context • Innovation design • Characteristics and perspectives of recipients of innovation • Characteristics and perspectives of individuals involved in delivery of innovation across multilevel roles • Implementation/sustainability infrastructure	• Papers describing PRISM, including updates over time [14, 32] • Re-aim.org website resources relevant to PRISM • Example interview guides asking about PRISM determinants
CFIR	• Inner/outer context • Characteristics and roles of individuals involved • Innovation characteristics • Implementation process	• Papers describing CFIR, including updates over time [10, 11] • cfirguide.org website, including examples of interview questions
HEIF	• Recipients • Innovation characteristics • Clinical encounter • Societal influence	• Papers describing HEIF and its application [23, 25]

The EPIS framework captures hypothesized determinants within and across levels of outer and inner context and across four key process phases [12, 13]. PRISM focuses on perspectives and characteristics of both those delivering and receiving an EBI; it also emphasizes the importance of implementation infrastructure and external environment [14–16]. PRISM identifies key contextual factors associated with well-established implementation outcomes of Reach, Effectiveness, Adoption, Implementation, and Maintenance/Sustainment (RE-AIM) [14–16]. CFIR, which was updated in 2022 based on user feedback, focuses on contextual factors associated with successful implementation [10, 11]. CFIR structures determinants across socioecological levels but does not depict interrelationships, whereas EPIS includes factors that bridge the outer and inner contexts and captures the characteristics of the innovation being implemented. PRISM’s implementation and sustainability infrastructure also spans contexts. The HEIF highlights contextual factors and their interactions (e.g., encounter between recipient and deliverer) that can influence implementation and disparities in healthcare [23].

### Framework crosswalk to create IM4Equity

The first step in the crosswalk process involved collecting and reviewing published papers and other relevant sources (e.g., framework-specific websites, example interview guides) identified through an online search and recommendations by the framework’s developers (Table 1). Throughout the review phase, authors LGP and ARD generated a list of all the domains and constructs of each framework, grouping those that overlapped and separating those that were unique to a framework. We then developed names and definitions for the identified domains and constructs, seeking to retain as much language as possible from the original framework(s) while incorporating new details and specifying areas of overlap. We documented this information in a comprehensive Excel spreadsheet (Supplementary Material 1).

To accompany the list of determinants, we created a prototype summary figure that visually depicted the domains. Within each domain, we list examples of key constructs from the literature as well as examples of factors that either maintain systems of oppression or contribute to health justice/equity. These examples are for illustrative purposes only; for instance, ‘structural racism’ is shown under Societal Context, but other factors may be included here that are more relevant to other populations of focus, such as ableism or transphobia. Unlike the source frameworks, which all depicted domains as separate, we sought to highlight the interconnections among domains by illustrating overlap among them where appropriate. We also created three simplified versions for specific settings represented in DECIPHeR Alliance projects (healthcare, education, and faith-based organizations) to show its applicability to diverse partnerships; the simplified figures only denote examples of equity-related factors to more clearly distinguish them from the main figure and avoid redundancy. Figure development was led by ARD, LGP, and a graphic designer. We also obtained informal initial feedback on the figure from the DECIPHeR Implementation Science Subcommittee.

### User-centered design feedback process

To ensure IM4Equity is usable for equity-focused research-community partnerships, we used a user-centered design (UCD) approach, also known as human-centered design. UCD places the intended user of a product at its center. It grew out of various fields such as psychology, industrial design, and human–computer interaction [33–35]. UCD includes a set of principles intended to make products more accessible, appealing, and usable [36, 37]: identifying intended users, involving real users in testing, collecting data from intended users, and making changes based on the data obtained.

Our UCD approach involved (a) identifying our intended users (i.e., researchers and community partners conducting implementation projects to promote health equity), (b) conducting focus groups in which we introduced the purpose of IM4Equity and demonstrated each of the domains and constructs, (c) gathering participant impressions, usability perceptions, and recommendations for improvements, and (d) iteratively refining the IM4Equity figure.

We applied two rounds of focus groups with DECIPHeR Alliance team members and their community partners. The first round focused on gathering feedback on the main figure and simplified setting-specific figure versions (healthcare, education, faith-based). The second round took place at an in-person Alliance meeting and focused on gathering feedback on additional materials developed in response to recommendations from the first UCD round, specifically for supporting community engagement in applying IM4Equity. Informed consent was waived for all participants by the RAND Institutional Review Board, which determined this work as exempt from full review.

For the first round, we asked the Principal Investigators/Multiple Principal Investigators from each of the seven DECIPHeR IRCs to nominate participants from their teams, including but not limited to a Principal Investigator, implementation researchers, qualitative and mixed-method researchers, community engagement and health equity experts, and representatives from community partner organizations. Individuals with these roles were identified because they are the primary (direct) and secondary intended users of IM4Equity. We conducted seven focus groups– one with each DECIPHeR IRC. A total of 43 participants provided feedback, including 38 researchers, 3 community partners, and 2 who identified as dual-agents (both researcher and community partner). We also received informal feedback from DECIPHeR’s Implementation Science and Community Engagement Subcommittees, the latter of which includes both researchers and community partners. We did not count the number or role of those in these Subcommittees as part of our sample size for the present analysis given presentations to these groups were for informational purposes and we welcomed questions or comments through an open-ended discussion (i.e., we did not use a structured protocol to gather specific input).

The first round of focus groups were held via Zoom between January 2023 and February 2024. One week ahead of each focus group, we e-mailed participants a draft of the latest IM4Equity figure, one of the simplified setting-specific figures (relevant to their project), and the project consent form. We sought to limit the size of each focus group to 6–9 participants [38].

Each focus group was led by a trained moderator (JLM) and an additional person (LGP, AGH, or ARD) assisted with fielding questions during/after the presentation. The focus group involved: a) introductions, b) reading the informed consent form, c) presentation on the rationale for and development process of IM4Equity, and d) an interactive walkthrough of the elements of the figure. Open-ended, qualitative questions developed by the co-authors for this study were asked to capture general impressions, what participants liked or did not like, and what they would like to see changed and why (Supplementary Material 2). Separate questions were asked of researchers and community partners to further assess how each group might approach using IM4Equity. Each focus group lasted 60 min and was audio-recorded for analysis. Community partners that the DECIPHeR teams invited were offered a $50 gift card for their participation; researchers supported by their DECIPHeR project did not receive payment.

The feedback from these focus groups informed the development of additional materials to guide community engagement in co-creating and applying IM4Equity (i.e., ensuring community partners participate in the identification, prioritization, and operationalization of determinants). These materials included a process flow chart, a blank IM4Equity template (with fillable domain and construct labels), and a guidance document walking through the steps for completing the blank template. We presented these additional materials to DECIPHeR Alliance team members at a 2-day in-person meeting in New Orleans, LA in May 2024. We invited meeting attendees to participate in a 90-min breakout session to review and provide feedback on the new materials. A total of seven individuals, representing four IRCs plus the RCC, attended the breakout session. We split the group into two and asked each smaller group to fill out the template using the guidance document for one of their own projects (i.e., user testing, a key UCD activity). We gathered feedback through a group discussion and brief survey. Given only 4 individuals submitted their surveys, we omit those results in this manuscript to prevent potential identifiability. We took notes on opinions and suggestions that arose during and after the breakout session, focusing on recommendations for improving the materials.

### Data analysis and iterative refinement of IM4Equity

We followed standard processes for rapid analysis of qualitative data [39–41]. The audio-recordings from the focus groups were tabulated into individual quotes in an Excel file. Each quote was coded by major feedback categories (e.g., general impression, recommendation, usability). We reviewed each comment and recommendation and determined how to address it.

After the first four focus groups were conducted, we met in June 2023 to review and incorporate the initial feedback, resulting in updated figures that were used in the final three focus groups. We met again in March 2024, after all focus groups were completed, after which we finalized the figures and drafted the supporting materials.

Finally, we reviewed the notes from the in-person breakout session focused on the IM4Equity supporting materials. LGP made a list of the suggestions and updated the content of the process flow chart and guidance document. The graphic designer reviewed and incorporated all recommended design suggestions to the supporting materials.

## Results

### Results from initial review of framework resources

The Excel spreadsheet documenting the final crosswalk of determinant domains and constructs from CFIR, EPIS, HEIF, and PRISM is found in Supplementary Material 1. We identified seven domains across the frameworks: 1) Recipient and Organization Perspectives on the Program/Practice, 2) Community Context and Recipient Factors, 3) Organizational Context and Personnel Factors, 4) Recipient-Deliverer Interactions, 5) Bridging Factors, 6) Implementation Process, and 7) Societal Context. Only the Recipient-Deliverer Interactions domain was unique to one framework (HEIF); it is called "clinical encounter” in HEIF, but we renamed it to be inclusive of interactions beyond clinical settings. The list of domains informed the structure of the initial prototype IM4Equity figure.

### Feedback from round 1 of UCD process

Table 2 summarizes the feedback and example quotes from the focus groups in the first round of UCD, which focused on the main IM4Equity framework and simplified setting-specific figures. Feedback was separated into nine categories that guided iterative refinement of the framework.

**Table 2:** Key feedback and quotes from the 7 focus groups with DECIPHeR Alliance research teams and their community partners

**Feedback categories**	**Example quote(s)**
General comment	“I like the name of the framework and the focus on equity.”
	“The framework is hard to understand at first, but the more I think about it, the more sense it makes.”
Recommendations for improving the design, interpretation, and/or readability of the main IM4Equity figure	“Highlight or draw more attention to the Key at the top of the figure.”
	“The figure comes across as very busy, consider increasing the font size and reducing the blank space”
Comments about IM4Equity usability or recommendations to increase usability	“This would be great for community engagement. People can pick and choose what pieces are most relevant to one’s project, but they don’t need to measure everything.”
	“It might be helpful to consider creating questions that would answer these and get at determinants, such as an interview guide.”
Recommendations or concerns about specific determinants in IM4Equity	“In recipient factors, mistrust in organization is an *outcome* of systems of oppression vs. being enacted on, which all the other examples of maintaining systems of oppression are of. Focus more on drivers vs. outcomes. Impacted communities can’t maintain systems of oppression.”
	“‘Bridging factors’ is one term that my community can understand but other terms seem too technical.”
Recommendation for improving the interpretation of one of the case example figures	“I don’t think I could make a distinction between faith community and faith institution. It would be helpful if the terminology could be more descriptive and clear.”
Factors necessary for use as part of community engagement	“Antiracist components need to be implemented with a significant level of trust in the community. Trust needs to be established– needs to be at the forefront.”
	“Communities need to be part of the process as self-conscious agents. Present it to them and get their stake in the game.”
Recommendations for improving the accessibility of the IM4Equity figure	“Guidance documents would be needed for it to be accessible by a community partner.”
Recommendation for additional support materials: Fillable Template	“It would be helpful to see a version with all of the bulleted text taken out, leaving just the domain titles. That way I could fill it in with what matters for my site or community”
Recommendation for additional support materials: Facilitation Guide	“For the facilitation guide, step 4, facilitator or discussion guideline should include versions tailored to those who are trying to facilitate a discussion. This can include different levels if folks aren’t framework savvy, such as a higher level collective sensemaking (light version) to lower the bar to starting these discussions.”

Participants identified a variety of strengths of the framework as well as areas for improvement. We worked with the graphic designer to iteratively update the IM4Equity figure and simplified setting-specific figures according to suggested edits. Changes included: reorganizing the layout of the domains to improve readability and interpretability, re-designing how temporality was displayed to specify EPIS phase, and simplifying language. Further, participants frequently identified a need for guidance materials, such as a blank template and a facilitation guide to use when engaging with community partners, which were developed and subsequently tested in the second round of the UCD process.

### Feedback from round 2 of UCD process

**Fig. 1:**
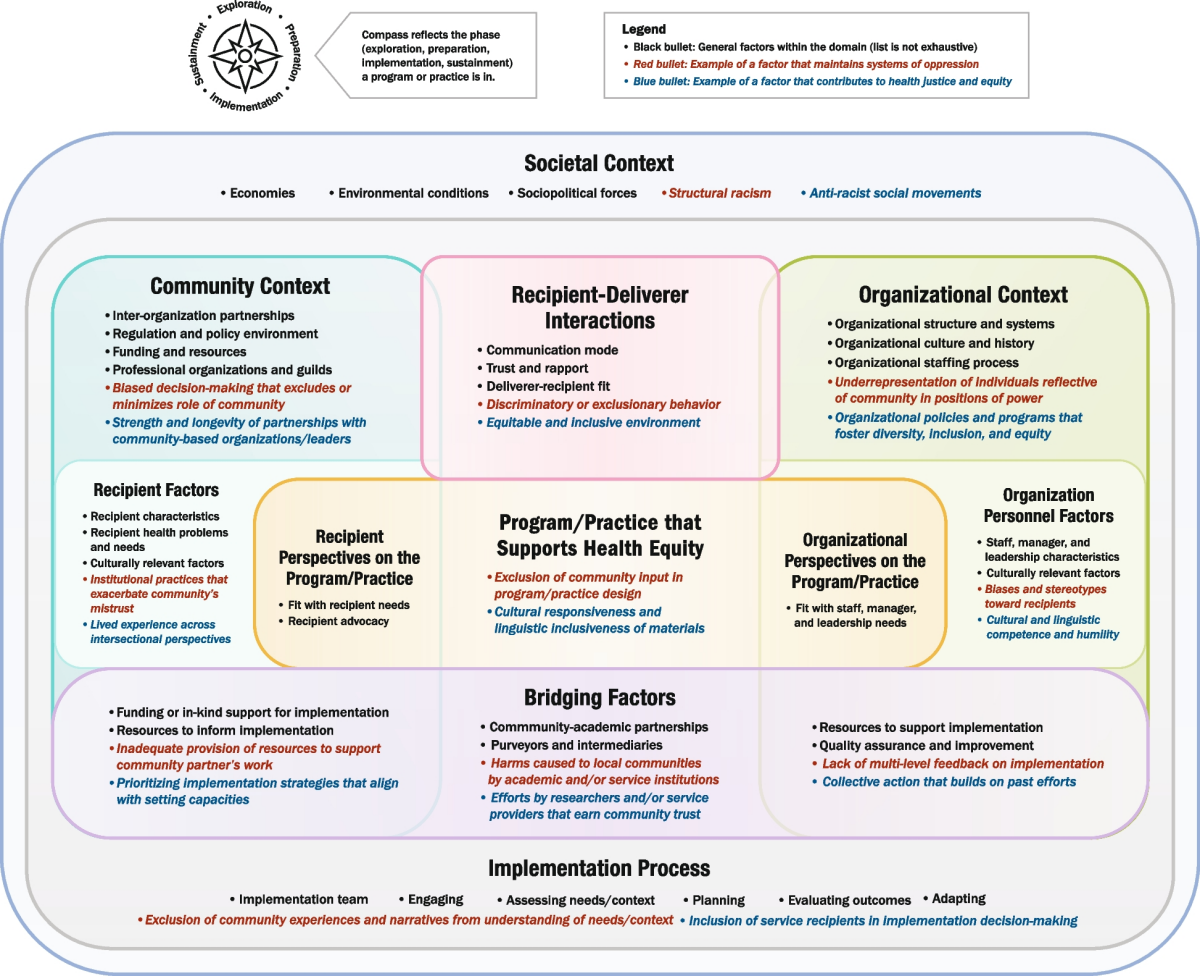
IM4Equity (Crosswalk of 4 IMplementation Frameworks to advance health Equity)

**Fig. 2:**
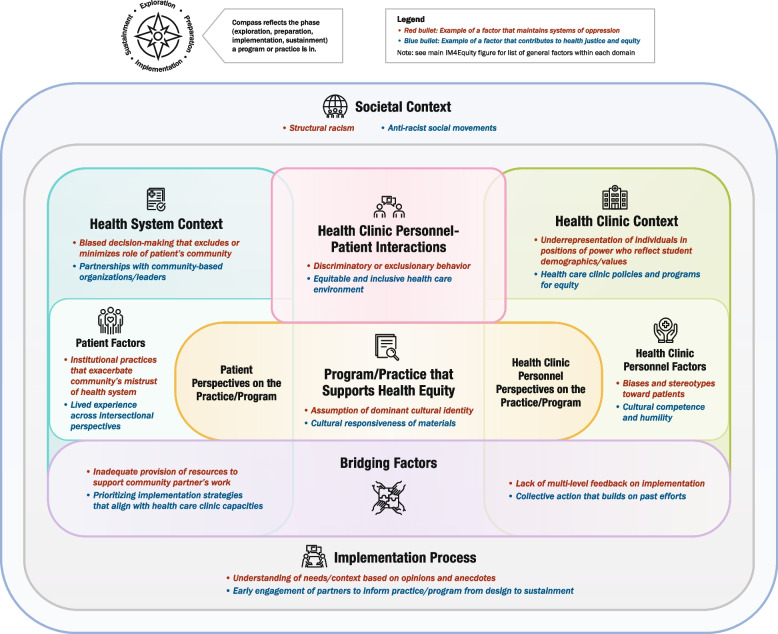
Healthcare-specific IM4Equity simplified figure

**Fig. 3:**
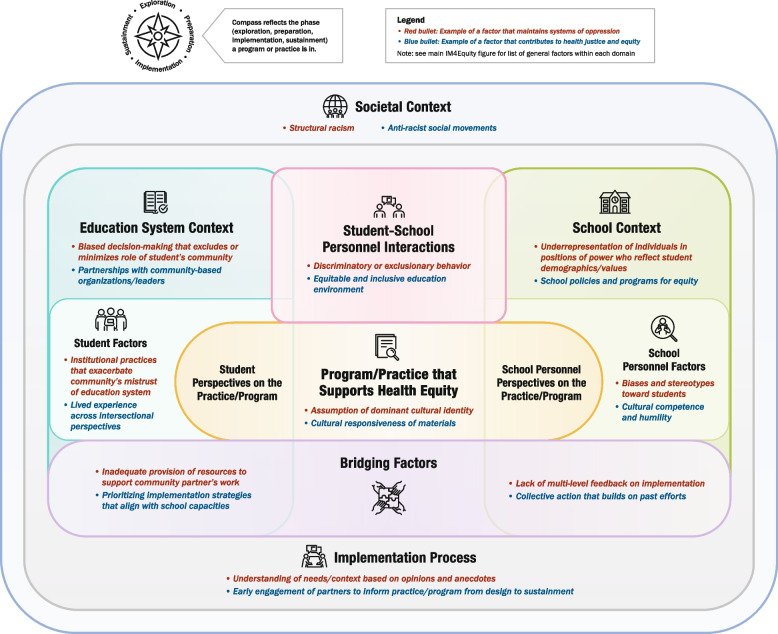
School-specific IM4Equity simplified figure

**Fig. 4:**
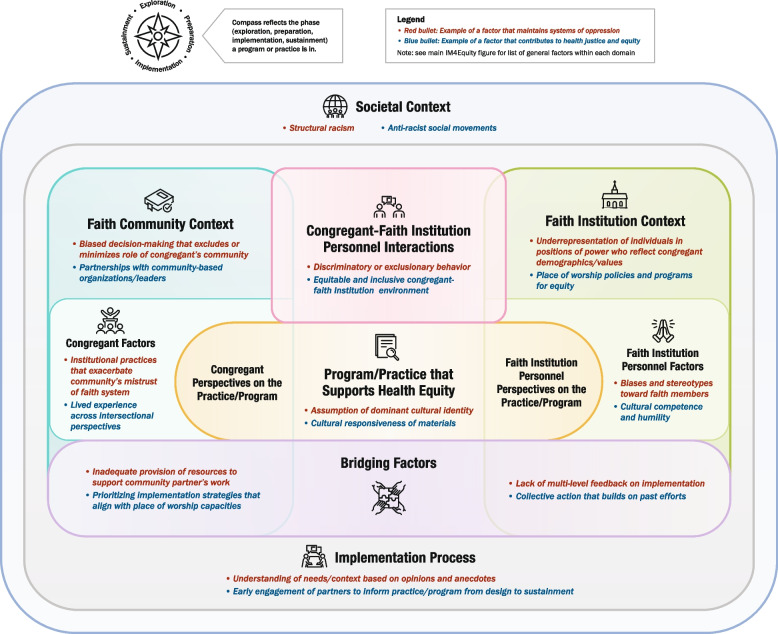
Faith-based setting-specific IM4Equity simplified figure

Participants of the in-person IM4Equity breakout session at the DECIPHeR Alliance meeting identified several recommendations for improving the initial drafts of the flowchart, figure template, and guidance document. For the flowchart, there was a suggestion to add clarification in step 1 that research-community teams should have identified the EBI and target health outcome before using IM4Equity; as well as emphasis on considering which EPIS phase the project is currently completing in step 4. For the IM4Equity template, participants recommended providing guidance on characteristics of the person who will be facilitating discussions with researchers and community partners to complete the template. There was also a suggestion to reorder the numbering of the domains to be more streamlined. For the template guidance document, recommendations included adding prompts for identifying the EPIS phase of the project; prompts to guide how one would prioritize constructs; and clarification on how long it may take to go through the process of completing the IM4Equity template for one’s project. Participants also suggested ensuring the labels were consistent across the materials. The deliverables from this second round of feedback included: 1) a final version of the IM4Equity figure (Fig. 1) and simplified setting-specific figures (Figs. 2, 3 and 4) and 2) updated supporting materials (Supplementary Materials 3, 4 and 5).

## Discussion

With key input from community and research partners in the DECIPHeR Alliance, we developed IM4Equity, an implementation determinant meta-framework that is one of the first to provide guidance on how to co-create and apply the framework with community partners for a specific EBI and context. IM4Equity may be used across the life cycle of community-engaged research to identify contextual- and equity-specific determinants of implementation of EBIs in diverse community settings. Feedback from Alliance team members led to the development of resources to promote community engagement in the application of IM4Equity to ensure determinants are operationalized in ways relevant and appropriate for the local context. By understanding common implementation and equity determinants with community input, IM4Equity may help promote shared learning in the Alliance and other multi-site research initiatives aimed to promote health equity.

Few theories, models, and frameworks for implementation science are equity-focused [4, 22, 23]. Thus, IM4Equity is a meta-framework that combines three of the most widely used determinant frameworks and a health equity-centered framework to call attention to cross-cutting domains of determinants with a focus on equity. IM4Equity is unique in highlighting a range of traditional and equity-specific constructs and domains to consider, including how they may vary at different implementation phases [10, 13, 42], and providing guidance on community engagement.

IM4Equity calls on researchers to meaningfully engage communities in identifying equity factors across every domain, thereby responding to multiple calls for better integrating equity in implementation science theories, models, and frameworks [18, 24, 25]. Our UCD approach of engaging researchers and their community partners to inform IM4Equity also highlights the importance and power of community engagement in advancing equity in the field of implementation science. The feedback collected inspired the creation of additional resources to guide IM4Equity’s application in practice, thereby improving its potential utility and acceptability among research-community partnerships. This may be particularly relevant for implementation practitioners and/or program evaluators who have limited implementation science expertise. It is also an initial step to build capacity for more community-led assessments of implementation determinants. The feedback we received also highlights that there is further work that can be done to co-produce materials to facilitate the use of IM4Equity and other frameworks, but this work was deemed an important step forward.

Given IM4Equity was developed with an ongoing multi-project Alliance in mind, it may be particularly useful for other research consortia that wish to understand implementation determinants from multiple frameworks, specifically EPIS, PRISM, CFIR, and HEIF. IM4Equity’s equity focus and adapted language to be more general (i.e., less clinical) also suggests it may be particularly relevant for community-engaged health equity projects implemented in diverse settings that have wide reach among populations that are socially disadvantaged (e.g., faith-based organizations). Further, IM4Equity is meant to be adaptable to fit a range of projects and partnerships. Thus, while some frameworks provide specific definitions for their constructs, IM4Equity promotes community engagement in the operationalization and measurement of constructs to ensure they are relevant and acceptable to the community that will be impacted by the EBI. Further, for projects that already started using a framework or a combination of frameworks, the guidance materials from IM4Equity on how to engage community partners in identifying equity-relevant determinants across implementation domains can still be of high value.

Results from the UCD process provided insightful and actionable feedback from our partners. Community participants inspired the creation of additional resources to increase IM4Equity’s utility. However, participants also noted the value of designing a figure that ‘stands alone’ and makes intuitive sense without needing several adjunctive resources. Therefore, we made concerted efforts to provide a visually appealing and intuitive framework that can be accessed alone as well as guidance on its use for those who want to drill down and consider the process of incorporating equity constructs and their relationships at granular levels. Moreover, an important equity consideration of our UCD approach was the ability to compensate community partners for their time and effort. Overall, the UCD process warrants patience, careful attention to various perspectives, understanding the power of language and being responsive to adapt terminology and definitions to resonate with different populations and contexts.

### Potential advantages and limitations of IM4Equity

IM4Equity has potential advantages and limitations as compared to other frameworks for community-engaged implementation initiatives. First, it provides guidance on engaging communities and promotes co-creation in informing and operationalizing implementation determinants with a pro-equity lens; this prompts users of IM4Equity to consider how determinants of equity relevant to their project will be defined, measured, and potentially addressed. Second, IM4Equity combines the different strengths of multiple frameworks and can help develop a more comprehensive understanding of key determinants and/or processes of implementation of health equity-focused EBIs. Third, the UCD approach led to feedback and recommendations to strengthen IM4Equity’s usability, readability, and acceptability across a range of potential users. The resultant recommendations for additional resources to facilitate application of IM4Equity inspired the development of supporting materials that can improve community partner engagement in applying IM4Equity, including individuals with limited implementation science expertise. These materials are key in guiding an inclusive approach to help community-engaged teams work together to adapt and apply IM4Equity for their specific needs.

In terms of limitations, feedback was obtained from a single alliance of implementation research centers (DECIPHeR) and fewer community partners participated in the UCD rounds compared to researchers. As such, the feedback we received from the community partners may not represent all community perspectives and opinions about IM4Equity. Despite the limited participation of community partners, we prioritized the community input to ensure their perspectives were incorporated as we refined IM4Equity. Importantly, the community feedback informed key changes that resulted in replacing scientific jargon with more user-friendly language as well as the creation of guidance materials for applying IM4Equity in diverse community settings, which has not been developed for previously published frameworks. Further, participants represented diverse personal and professional backgrounds as well as diverse perspectives based on their roles on the projects; a range of expertise levels in implementation science (from little to high); a range of health outcomes of focus; and diverse geographic and implementation settings (healthcare, education, faith-based). As such, the diverse perspectives may reflect those of other similar community-based projects across the country. Nevertheless, a larger sample of community partners may have elicited additional areas that were not considered. Further, use case testing is needed by other research-community partnerships to understand how it is used, what adaptations are made, and to generate external validity evidence. Ongoing work is needed to continue to enhance IM4Equity and refine the supporting materials to strengthen its utility and impact in the implementation science field and health equity. We encourage those who use IM4Equity in their work to provide feedback to the authors on what works and areas for improvement.

The simplified setting-specific framework examples focused on three settings (healthcare, education, faith) that are of focus in the DECIPHeR Alliance. However, these settings are not generalizable to other community contexts, such as criminal justice. These examples are only intended to help teams visualize how they might adapt IM4Equity for their context, with the option of using the guidance document (Supplementary Material 5) to help fill out the blank template of IM4Equity (Supplementary Material 4).

In addition, the crosswalk was limited to four frameworks. There are many existing theories, models, and frameworks that may have constructs not reflected in IM4Equity. Thus, IM4Equity may need further adaptation to best fit different project needs. Alternatively, one may choose to use their preferred implementation framework and focus on the guidance materials provided here for engaging communities and co-creating their implementation framework with their community partners. Despite adaptations to the terminology in IM4Equity, the words may still be too technical and thus require additional adaptation and operationalization by those using IM4Equity, as supported by the template and guidance. Further, IM4Equity does not focus on implementation outcomes and thus, additional evaluation frameworks (e.g., RE-AIM) may need to be used, or perhaps even integrated into future revisions given that equity considerations are also highly relevant to implementation outcomes. Finally, IM4Equity does not focus on mechanisms by which determinants contribute to implementation outcomes.

## Conclusions

IM4Equity is a meta-framework that highlights overlap among existing frameworks. It presents a comprehensive set of determinants and is one of the first frameworks to promote co-creation by ensuring community partners participate in the process of identifying, prioritizing, and operationalizing the most relevant and appropriate determinants for one’s context, including both implementation *and* equity facilitators and barriers. The supporting materials we developed highlight the general approach one would use to apply IM4Equity for community-engaged projects. These resources will allow for a more community-centered approach to specifying IM4Equity in a way that promotes inclusivity and shared ownership, and could be adapted for use with other determinant frameworks. To continue to advance efforts to support community engagement in the identification and operationalization of determinants for the field at large, we strongly encourage others to also develop supporting materials to promote community engagement in the identification and operationalization of determinants. Such collective efforts are critical to ensure that community partners are engaged in the process of identifying what constructs are most relevant to the cultural and structural contexts of their community and are imperative to advancing health equity.

## Supplementary Information

Supplementary Material 1.

Supplementary Material 2.

Supplementary Material 3.

Supplementary Material 4.

Supplementary Material 5.

## Data Availability

Data presented in this article are not available given they cannot be deidentified.

## Abbreviations

CFIR: Consolidated Framework for Implementation Research
DECIPHeR: Disparities Elimination through Coordinated Interventions to Prevent and Control Heart and Lung Disease Risk
EBI: Evidence-based innovation
EPIS: Exploration Preparation Implementation Sustainment
HEIF: Health Equity Implementation Framework
IM4Equity: Crosswalk of 4 IMplementation Frameworks to advance health Equity
IRC: Implementation Research Center
PRISM: Practical Robust Implementation and Sustainability Model
RE-AIM: Reach, Evaluation, Adoption, Implementation, and Maintenance
UCD: User-centered design
