# Supplementary material for: IM4Equity: an implementation science meta-framework for community-engaged partnerships to advance health equity
Source: BMC Health Serv Res. 2025 Mar 26;25:437. doi: 10.1186/s12913-025-12537-8 (PMC11948705; doi:10.1186/s12913-025-12537-8)
Supplement: Supplementary file 2 — Supplementary Material 2. [file 12913_2025_12537_MOESM2_ESM.docx]

EPIS-PRISM-HEIF-CFIR 2.0 crosswalk feedback

Discussion script for capturing input from research teams/community members

**Feedback area #1: show GRAPHIC of framework crosswalk (slide deck or PDF) with a 1-page set of talking points about key factors that influence the success of implementation**

Talking points to set up the presentation to a community audience ‘on tour’:

- **Who we are**: an alliance of 7 research teams across the US who are seeking to reduce cardiopulmonary inequities by delivering evidence-based programs.
- **What is the purpose of today’s discussion**: we have created a visual framework to help us share our challenges and lessons learned as we plan and conduct our studies. We developed this visual to be able to guide research teams in assessing common determinants and compare findings to enhance understanding of what influences effective intervention implementation across diverse contexts.
- **How have we developed this visual? What does it show:** This visual integrates four frameworks that describe the factors that influence the success of implementing evidence-based programs into real-world settings.
  - We identified a common set of determinants across the three frameworks being used across our alliance of 7 research teams, and that describe the influences of contextual factors upon implementation: the Exploration, Preparation, Implementation and Sustainment (EPIS) framework, the Pragmatic Robust Implementation and Sustainability Model (PRISM), the Consolidated Framework for Implementation Research version 2.0 (CFIR 2.0). Given that EPIS and PRISM do not explicitly call out equity-specific influences, we also layered on elements of the Health Equity Implementation Framework (HEIF), in order to capture factors that may particularly influence the successful implementation of programs designed to reduce disparities in communities that experience an inequitably high burden of chronic disease.
  - You can see that the graphic shows around the outside a set of implementation phases that occur, moving from Exploration to Preparation to Implementation to Sustainment. However, this is not a linear process, it’s more dynamic; one can think about sustainment early on or use learnings from one phase to go back to a previous phase and adapt as needed
  - The graphic itself has ‘layers’ like an onion that move from right to left from the ‘Inner setting’ where a program is actually implemented – and then moves leftward towards the recipients of the program.
  - Moving further leftward, it shows the outer setting and external environment that is relevant to the recipients and delivers of this program, in terms of external influences on how the program is implemented, whether there are influences from the policy realm or influences in other ways.
  - At the top of the diagram are a broader set of external factors such as societal forces.
  - At the bottom of the diagram, we see a set of factors that influence the sustainability of a program that span the full spectrum of inner setting to outer setting, and that include bridging factors between the inner setting and outer setting.

**TRANSITION TO DISCUSSION:** Before this transition, for the researcher group only, mention there is a complementary file w/ examples of each construct so teams can identify specific things to measure under each bucket. We’ll discuss after the visual.

1. Overall impression of the graphic – what do you think?
   1. What do you like about how this shows factors that influence the implementation of a practice or program?
   2. What do you find hard to follow or confusing?
2. What would you like to see improved about this visual? And suggestions for how to improve it?
   1. Probe: graphic representation (e.g., too much text/too little text or explanation, color coding), language (too academic/confusing, etc.)
3. In terms of the red font and blue font items that relate to health equity – do you have any particular comments about these?
4. In what ways do you see your research team using this visual as you plan or evaluate a project?
   1. Think aloud with us – are there any specific ways you could see using this visual as part of a community advisory board or other community-engaged meeting for your project? If you were using this type of visual in that setting, what would you do with it?
      1. RESEARCH TEAM PROBE – Are there ways we could improve this visual for explaining aspects of your project to your community?
      2. COMMUNITY PROBE does this visual help you to see the types of factors that would lead to the successful delivery of a research program to improve health disparities in your community?
         1. How doable would it be for us to use this graphic to start a community discussion about those types of factors that may influence implementation of an innovation?
         2. In what way would you see community members appreciating the elements outlined on this visual – finding them useful? In what ways would this not be useful?
         3. What if there were specific examples for each of these elements from their own community that were called out?
         4. What aspects of this would be too complex or pose a stumbling block to review and discussion with a community audience?

**Feedback area #2: EXCEL file for RESEARCHER REVIEW** (not sure this makes sense for community)

Talking points: we developed this file to identify specific example measures within each of the elements of the visual diagram that we just talked through. The idea is that this helps us to identify things to measure and assess and to clarify if the domain is classically represented in the EPIS, PRISM, and/or HEIF

1. Overall impression of the Excel file – after being introduced to the visual crosswalk, does this intuitively pair with that visual? Are there aspects that don’t make sense from an organization standpoint?
2. Review the domains for the inner setting – any specific questions or comments?
3. Review the domains for the outer setting – any specific questions or comments?
4. Repeat for each area of the Excel file
5. Think about using this for your DECIPHeR project, but NOT having to fill in every single row and just using it as appropriate --- in what way would you see yourself using it in the planning phase? In the implementation phase? In the evaluation phase?
   1. what would be beneficial about using it?
   2. What would be painful?
   3. What improvements would you like to see in this document?
