# Supplementary material for: IM4Equity: an implementation science meta-framework for community-engaged partnerships to advance health equity
Source: BMC Health Serv Res. 2025 Mar 26;25:437. doi: 10.1186/s12913-025-12537-8 (PMC11948705; doi:10.1186/s12913-025-12537-8)
Supplement: Supplementary file 3 — Supplementary Material 3. [file 12913_2025_12537_MOESM3_ESM.pdf]

1 Decide to use IM4Equity

- IM4Equity helps community-engaged research teams to document factors affecting implementation of programs and practices that support health equity
- Before using IM4Equity, the health equity topic and program/practice should already be identified and informed by the evidence base and community input, including acceptability and feasibility

2 Select team members

- It is essential that team includes perspectives of community partners and program/practice recipients
- Having implementation science expertise is ideal, but this can be limited to one or a few members

3 Get oriented to support materials

- The detailed framework graphic is most useful for implementation researchers
- Case example graphics show how IM4Equity could be customized to projects based in healthcare, education, and faith settings

4 Fill out the template

- Team members from Step 2 fill out the template with factors/language tailored to their specific context and considering what phase the program/practice is in (exploration, preparation, implementation, or sustainment)
- The template includes brain-storming outlines and example factors to get teams started

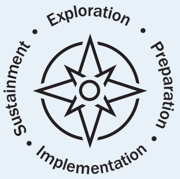

5 Monitor and update

- Review/update the template at each new project phase
- Add or replace team members as appropriate; be sure to maintain community partner/recipient representation
- Revisit previous decisions if needed – this is an expected part of the process!

Additional steps after IM4Equity

- Choose and use measures to monitor factors identified
- Select strategies for supporting implementation that are tailored to strengths and needs
- IM4Equity does not include materials to complete these steps, but it can inform them
