# Supplementary material for: IM4Equity: an implementation science meta-framework for community-engaged partnerships to advance health equity
Source: BMC Health Serv Res. 2025 Mar 26;25:437. doi: 10.1186/s12913-025-12537-8 (PMC11948705; doi:10.1186/s12913-025-12537-8)
Supplement: Supplementary file 4 — Supplementary Material 4. [file 12913_2025_12537_MOESM4_ESM.pdf]

Compass reflects the phase (exploration, preparation, implementation, sustainment) a program or practice is in.

Date: ____________________

7 Factors in Society

2 Community Factors

2a. Community context

2b. Community member factors

Community views

4 ______________ Member-Personnel Interactions

ADD LABEL FROM 2B

____

ADD LABEL FROM 3B

3 Organizational Factors

3a. Organization context

3b. Personnel factors

Personnel views

1 Program/Practice and Health Equity Topic

5 Bridging Factors

Supports in the community

Connections between supports

Supports at the organization

6 Process Factors
