# Supplementary material for: IM4Equity: an implementation science meta-framework for community-engaged partnerships to advance health equity
Source: BMC Health Serv Res. 2025 Mar 26;25:437. doi: 10.1186/s12913-025-12537-8 (PMC11948705; doi:10.1186/s12913-025-12537-8)
Supplement: Supplementary file 5 — Supplementary Material 5. [file 12913_2025_12537_MOESM5_ESM.docx]

**Instructions for Completing the IM4Equity Template**

**Why use IM4Equity?**

1. IM4Equity is a meta-framework that can help identify a range of factors that facilitate or impede successful implementation of an evidence-based innovation (EBI; a specific program or practice) aimed to promote health equity in a community. It is a “meta-framework” because it highlights domains and specific factors within those domains that have been identified by multiple existing frameworks (EPIS, CFIR, PRISM, HEIF*) to play an important role in the successful implementation of a program/practice, such as the individuals involved in delivering the program/practice, characteristics of the community intended to receive the program/practice, and the broader context in which a program/practice takes place.
2. By engaging community partners and researchers in collaboratively identifying and understanding how multiple factors influence program/practice implementation, the information can be valuable in guiding program/practice translation to other communities and inform sustainability for broader and long-lasting impacts on health equity.

**Why use the IM4Equity template?**

1. This template is a simplified version of the IM4Equity main figure. Community-research partnerships can tailor it for their specific program/practice, including identifying and documenting specific factors that the partnership team deems relevant and most important to their context. The template is designed to be flexible and locally informed so that partnership teams can select the domains and factors most relevant to their settings and populations as well as tailor the labels and definitions of those factors to ensure cultural appropriateness.
2. Completing the template may include identifying factors for all 7 domains, this may help produce a rich understanding of facilitators or barriers to implementing a program/practice; however, the partnership team may instead choose to focus on a few domains of higher priority to their work or community.
3. The template can be updated across the lifespan of a program/practice (e.g., needs assessment, planning, implementation, sustainability) to ensure the factors remain relevant to the stage the program/practice is in.

**What happens after the template is completed?**

1. Once the partnership team has completed the template and are satisfied with the identified factors (including how they are defined), consider ways in which it can be applied, such as developing a plan to measure the identified factors and/or selecting strategies or approaches to address those factors.

*Footnote: EPIS = Exploration, Preparation, Implementation, Sustainment framework; CFIR = Consolidated Framework for Implementation Research; PRISM = Practical, Robust Implementation and Sustainability Model; HEIF = Health Equity Implementation Framework

To guide community-research partnership teams in using the IM4Equity template, we recommend the following steps across three phases: learning (**Get Ready**), pre-completion (**Get Set**), and completion (**Go**).

**Get Ready**

1. ***UNDERSTANDING IM4EQUITY***. To start using the IM4Equity template, please review the following resources:
2. Main IM4Equity figure – **Figure 1** shows 7 domains, with example factors within each domain (general factors, factors related to health equity, and factors that can maintain systems of oppression). The EPIS compass rose is intended to help teams first consider what phase their program/practice is in (exploration, preparation, implementation, or sustainment) given factors in each domain could vary by phase.
3. Setting-specific figures – **Figures 2-4** show what IM4Equity might look like for hypothetical healthcare, school, and faith-based partnerships. These figures can help teams see how they might want to complete their template for their setting. These setting-specific figures and the main figure can help ensure everyone is on the same page about what the broader domains are capturing and how the factors within those domains are related to health equity. In these setting-specific examples, the bulleted factors in each domain are only intended to spark conversation and teams are encouraged to go beyond these factors to identify what is most relevant and meaningful to their program/practice and community.
4. List of example factors - this spreadsheet (**Additional file 1**) lists examples of general factors for each domain (identified from the literature) and other equity-related factors to consider as you complete your template. This list is not exhaustive.
5. IM4Equity process flowchart – this flowchart (**Additional file 2**) provides an overview of the key steps for filling out the IM4Equity template (**Additional file 3**). Complete the first three steps before starting to fill out the template:
   1. Decide if IM4Equity can help obtain information that can meet your program/practice needs.
   2. If there is consensus on using IM4Equity, engage a diverse team from your partnership to collaborate in completing the template.
   3. As a team, review all IM4Equity figures and supporting materials to ensure everyone understands what the broader domains are capturing and how the factors within those domains are related to health equity.
6. ***SELECTING DISCUSSION LEADERS***. Identify a facilitator that will lead the partnership team in discussions for completing the IM4Equity template, as well as a notetaker to track ideas and key decisions made. Important characteristics of a facilitator may include (but are not limited to):
   - Experience leading group discussions, such as focus groups and breakout sessions.
   - Strong organizational skills and able to present as their authentic selves.
   - Strong communication skills, including being comfortable speaking in front of audiences; practicing active listening; paying attention to body language (e.g., when someone is excited or uncomfortable); keeping the conversation constructive and focused on a healthy balance of positives and negatives; and paraphrasing ideas to get clarity and/or validate individual’s comments as well as summarize and reiterate key themes, ideas, and decisions to create closure.
   - Flexibility to change the meeting structure to promote engagement, such as incorporating icebreakers and exercises that foster team bonding. If a conversation goes off topic, the facilitator must be patient and respectful of what is being shared while attempting to bring the group back to the topic of focus.
   - Neutrality while acknowledging differences in opinions among the partnership team. Facilitator may pose questions to help the group reflect on alternative opinions rather than dismissing different perspectives.
7. ***PLANNING DISCUSSIONS***. To complete the IM4Equity template, the partnership team may consider setting aside a few hours or breaking it up into multiple sessions to allow for sufficient time to support meaningful discussion, filling out the template, and decisions about next steps. Here is one example outline of the discussions:
8. Session 1 (1 hour): Review the IM4Equity figures and all supporting materials and assess how they will help identify key implementation and equity determinants of the EBI of focus to address the health problem the team is concerned about. Discussion may include reviewing definitions and understanding of the domains. This may help identify which domains to focus on for completing the template next.
9. Session 2 (1.5-2 hours): Engage a diverse group of team members (diverse in backgrounds and perspectives) to fill out the template, following the recommended order of the domains (1 through 7).
10. Session 3 (1 hour): Review the completed template and decide on next steps, such as selecting which determinants will be prioritized and how the determinants will be addressed, such as measurement (quantitative or qualitative), selecting implementation strategies to target those determinants, etc.

**Get Set**

1. Save or print a copy of the IM4Equity Template (**Additional file 3**) to fill out. A saved electronic version will be easier to review and update in the future. Add today’s date at the top of the page to remind the team when the template was completed (for historical records).
2. It’s important to document and acknowledge everyone who will contribute to the completion of the Template. Please fill out the following table with the names of those who will participate in discussions to inform the Template, including their role on the partnership team (e.g., Community Advisory Board member; Principal Investigator; Patient representative) and the communities they represent (e.g., faith community, research, patients with hypertension).

| **IM4Equity template contributors** | | |
| --- | --- | --- |
| **Name** | **Role** | **Communities Represented** |
|  |  |  |
|  |  |  |
|  |  |  |
|  |  |  |
|  |  |  |
|  |  |  |
|  |  |  |
|  |  |  |
|  |  |  |
|  |  |  |

1. Next, the identified team should **decide what EPIS phase your program/practice is in.** Table 1 describes the four phases and provides examples to guide your selection. Once you have made a decision, CIRCLE the selected phase from the compass rose at the top of the IM4Equity Template.

**Table 1.** EPIS phases and examples.

| **Phase** | **Description** | **Example considerations that could be expected in this phase** |
| --- | --- | --- |
| Exploration | Your team is in the process of choosing what program/practice to deliver, where, to whom, etc. For example, your team may be conducting a needs assessment, figuring out what intervention best fits your community. | - You’re identifying and understanding a clinical or health problem, including which populations are affected and what factors are driving the problem. - You’re searching for an EBI for addressing the clinical/health problem. |
| Preparation | Your team is getting ready to adopt the selected program/practice in an organization or community. For example, your team may be planning the intervention or conducting outreach to engage community interest to participate. | - Your team has selected an EBI and are planning its’ development and implementation (establishing funding, community outreach, partnerships, training, staffing, resources, etc.). - You’re deciding what, if any, adaptations to the EBI are needed, while monitoring fidelity to ensure changes do not compromise effectiveness. - You’re focused on understanding barriers and facilitators that influence implementation. - You have established clear goals and outcomes for the EBI. |
| Implementation | Your team is in the process of adopting and delivering the program/practice in an organization or community. For example, you are implementing the program/practice with organizational leadership support and sufficient resources. | - You’re implementing the EBI, while addressing implementation barriers/facilitators. - You’re evaluating outcomes along the way (e.g., health, process, implementation outcomes). - You’re monitoring implementation, fidelity, and feedback systems, and resolving challenges along the way. |
| Sustainment | Your team is maintaining delivery of the program/practice over time. For example, the program/practice is now part of the organization’s routine services and has stable support and monitoring. | - The EBI is engrained in the organization (or community), including stable funding and ongoing monitoring and/or quality assurance checks. - You’re focused on understanding factors that support or impede sustainment (e.g., additional resources, training, partnerships). - The organization that implements the EBI leads fidelity monitoring. |

1. Keeping the EPIS phase you selected in mind, now we’re ready to start **customizing the labels** **for the** **7 domains,** which are listed in Table 2, in the recommended order. Customize the labels so they are relevant and appropriate for your specific program/practice. Do not worry about the specific factors that will go under each domain, as that will be completed in the next (“Go”) phase.
2. Use the guidance in **Table 2** to help fill out the **bolded black underlines** in the **Template**. The colors of the table match those in the Template.

**Table 2.** Seven domains of IM4Equity and examples.

| **Domain** | **Filling out the black underline** | **Examples** |
| --- | --- | --- |
| 1. **Program/ Practice and Health Equity Topic**   [Center domain] | Write the name (or acronym) of the **program/practice** that will be implemented and the **clinical or health problem** the program/practice is addressing (if not mentioned in the program/practice name) | - LA Hypertension Equity Initiative |
| 1. **Community Factors ^a^**   [Top left domain] | Write in **2a)** the **community context** or larger system in which the program/practice will be implemented and **2b)** the intended **community members** to receive the program/practice. | - Generally, community context (2a) refers to a larger organizing network that guides or oversees the work of specific groups or organizations, e.g., a *diocese* oversees congregations and a *coalition* guides community-based organizations. This can also include systems at any level (local, state, federal, etc.), such as a district education system, county safety-net health system, local Veteran’s Affairs, etc. Do not mention the specific groups or organizations (e.g., churches, community-based organization, schools, clinics) that the community context/system oversees/guides, as they will be named in domain (3). - Examples of community members (2b) are patients, students, parishioners, etc., who could directly benefit from the program/practice. Be specific where appropriate (e.g., patients with high blood pressure). |
| 1. **Organizational Factors ^a^**   [Top right domain] | Write in **3a)** the **organizational** **context** in which the program/practice will be implemented and delivered and **3b)** the main **personnel** from that context who will be involved in implementing the program/practice. | - Generally, organizational context is the specific group or organization(s) in the *community context* defined in domain (2) that will be actively involved in carrying out the program/practice. Examples of organizational context (3a) include family medicine clinics, middle schools, food pantries, YMCA’s, etc. - Examples of personnel (3b) from the identified organizational context are health care teams, school staff, health coordinators/ministry, etc. |
| 1. **Community-Organization Interactions**   [Top center domain between domains 2 and 3] | Write in the same labels identified for domains 2b (community members) and 3b (personnel) above. | - For example, the label for a clinic-based program might be “Patient-Clinic Personnel Interactions” - For a school-based program, the label might read “Student -School Personnel Interactions” |
| 1. **Bridging Factors**   [Second to bottom domain] | No specific label is needed here. | - Supports at the organization fall under Organizational Factors and are internal people, resources, etc. that support implementation - Supports in the community are outside the organization and fall under Community Factors; these are external resources, people, etc. that support implementation - Connections among supports, especially those that connect supports inside and outside the organization, go in the middle |
| 1. **Process Factors**   [Very bottom domain] | No specific label is needed here. | - Examples of factors to list here include anything about how the implementation process has been approached, across implementation phases; this includes the team and activities involved (or not) |
| 1. **Societal Context**   [Most outer domain] | Write in the **broader context** that encompasses larger social, systemic, or structural factors that can influence the program/practice. | - Examples of societal contexts are the name of a country (if the program/practice is at a national level), region (e.g., Southern US), name of a state, or specific neighborhood or community. - Although a program/practice can be influenced by contexts across multiple levels (e.g., local, state), focus on the level(s) that might have the greatest influence on the program/practice. |

^a^ There is much diversity in health equity partnerships and teams are encouraged to adapt the domain labels to best fit their needs. For example, if your team wants to focus on the *dynamics between a community partner and research partner*, you can name the community partner organization and community members in domain 2 and the research institution and research team in domain 3.

1. To ensure your team is on the same page, come up with a brief description using the labels you created in the step above. For example:

*“This project is implementing [program/practice] to support [health equity topic] among [community members] served by [organization(s)] within [community context/system] in [societal context].”*

**Go!**

1. ***PHASE SELECTION***. Before discussing what factors will go in each of the seven domains of IM4Equity, keep in mind what phase (exploration, preparation, implementation, sustainment – see Table 1 on pages 4-5) your team identified in the “Get Set” phase (step 3). This will guide what factors will be most relevant to the discussion.
2. ***BRAINSTORMING***. Now your team is ready to identify specific factors in each domain of the template. We recommend the facilitator use the **optional brainstorming outline** on the following pages for each domain to help guide the selection of factors.
   1. The **brainstorming outline** includes prompts and questions to guide conversations about how to select and prioritize factors.
   2. For each of the seven domains (or group of domains if not all will be filled out), identify factors *most relevant* to the program/practice and communities of interest. We provide a list of example factors (**Additional file 1**) to get you started but teams are encouraged to tailor and go beyond these examples.
   3. When identifying factors for each domain, consider both strengths (facilitators) and needs or barriers. Write down the identified factors on a separate sheet or in the tables provided in the brainstorming outline. Do not fill out the Template yet.
   4. Throughout the brainstorming session(s), consider how the factors identified might contribute to health equity.
3. ***PRIORITIZATION***. After the team identifies multiple factors for each domain, the facilitator can lead conversations about which factors to prioritize. We recommend prioritizing factors your team believes may have the greatest impact on the program/practice’s implementation and are modifiable (via implementation strategies or other feasible changes in the organization or community). The prioritized factors can be added to the IM4Equity Template (in the colored underlines for the relevant domains).
4. ***UPDATING***. Revisit the Template periodically throughout the lifespan of the program/practice (i.e., from the start to end), and make updates as needed to reflect what your team is learning. This may mean updating the factors to align with the next EPIS phase the program/practice is in.

**Questions?**

Feel free to contact us at [adopp@rand.org](mailto:adopp@rand.org) with any questions about using IM4Equity. We will be happy to help support your use of this tool.

**
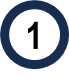

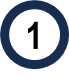
Program/Practice and Health Equity Topic Domain:**

**Brainstorming Outline**

**[see page 10 for instructions]**

**Domain description**: This domain is the yellow oval at the center of the IM4Equity framework. The factors for this domain will reflect characteristics of the program or practice that can facilitate or impede successful implementation, including how the program or practice is viewed by **A)** the community members intended to benefit from the program/practice (left side of yellow oval) as well as **B)** the personnel involved in its delivery at the implementing organization (right side of yellow oval).

1. **Community member views on the program/practice:**

| Factor (name, brief description or definition) | How factor relates to health equity (positive or negative) | Prioritize? ^a^ |
| --- | --- | --- |
|  |  | [ ] Yes  [ ] No |
|  |  | [ ] Yes  [ ] No |
|  |  | [ ] Yes  [ ] No |
|  |  | [ ] Yes  [ ] No |
|  |  | [ ] Yes  [ ] No |

Notes: Feel free to add more rows as needed.

^a^ We recommend prioritizing factors your team believes may have the greatest impact on the program/practice’s implementation and are modifiable (via implementation strategies or other feasible changes in the organization or community).

***Brainstorming questions to help identify factors:***

- What might be the community’s general thoughts of the program/practice (what might they know/not know about it and its potential impacts on the health topic in questions)?
- What factors could the community (the intended recipients of the program/practice) see as important to ensure the program/practice aligns with the needs of the community (e.g., language)? What might reduce alignment?
- What other characteristics of the program/practice could the community see as potentially influencing (positively or negatively) the program/practice’s impacts on health equity? [e.g., complexity of the program; for more examples, see **Additional file 1**]
- Which identified factor(s) does the team want to prioritize (e.g., assess or address in the future)?

1. **Organizational personnel views on the program/practice:**

| Factor (name, brief description or definition) | How factor relates to health equity (positive or negative) | Prioritize? ^a^ |
| --- | --- | --- |
|  |  | [ ] Yes  [ ] No |
|  |  | [ ] Yes  [ ] No |
|  |  | [ ] Yes  [ ] No |
|  |  | [ ] Yes  [ ] No |
|  |  | [ ] Yes  [ ] No |

Notes: Feel free to add more rows as needed.

^a^ We recommend prioritizing factors your team believes may have the greatest impact on the program/practice’s implementation and are modifiable (via implementation strategies or other feasible changes in the organization or community).

***Brainstorming questions to help identify factors:***

- What might be the organizational personnel’s general thoughts of the program/practice (what might they know/not know about it and its potential impacts on the health topic in questions)?
- What factors could the organizational personnel see as important to ensure the program/practice aligns with *their* needs (e.g., can easily fit in their current workflow or is it simple/complex; for more examples, see **Additional file 1**])? What might reduce alignment?
- What factors could the organizational personnel see as important to ensure the program/practice aligns with *supervisor and manager* needs? What might reduce alignment?
- What factors could the organizational personnel see as important to ensure the program/practice aligns with *site leadership* needs (e.g., addresses organizational priorities)? What might reduce alignment?
- What other factors could the organizational personnel see as potentially influencing (positively or negatively) the program/practice’s appropriateness or acceptability among the organizational personnel?
- Which identified factor(s) does the team want to prioritize (e.g., assess or address in the future)?

**
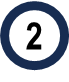

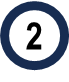
Community Factors Domain:**

**Brainstorming Template**

**Domain description**: This domain is the turquoise section on the left of the IM4Equity framework. For this domain, factors reflect the characteristics of the **A)** broad community context or system in which the program/practice will be implemented and **B)** the community members to receive the program/practice. This is not the specific groups/organizations that will implement and deliver the program/practice, as they are the focus of domain 3 (organizational factors).

1. **Community Context factors:**

| Factor (name, brief description or definition) | How factor relates to health equity (positive or negative) | Prioritize? ^a^ |
| --- | --- | --- |
|  |  | [ ] Yes  [ ] No |
|  |  | [ ] Yes  [ ] No |
|  |  | [ ] Yes  [ ] No |
|  |  | [ ] Yes  [ ] No |
|  |  | [ ] Yes  [ ] No |

Notes: Feel free to add more rows as needed.

^a^ We recommend prioritizing factors your team believes may have the greatest impact on the program/practice’s implementation and are modifiable (via implementation strategies or other feasible changes in the organization or community).

***Brainstorming questions to help identify factors:***

- How could partnerships the community context has with other organizations influence uptake of the program/practice?
- How could the regulatory environment and policies of the community context influence uptake of the program/practice?
- How could the funding and resources available in the community context influence uptake of the program/practice?
- What other factors in the community context could potentially influence (positively or negatively) the program/practice’s impacts on health equity?
- Which identified factor(s) does the team want to prioritize (e.g., assess or address in the future)?

1. **Community member factors:**

| Factor (name, brief description or definition) | How factor relates to health equity (positive or negative) | Prioritize? ^a^ |
| --- | --- | --- |
|  |  | [ ] Yes  [ ] No |
|  |  | [ ] Yes  [ ] No |
|  |  | [ ] Yes  [ ] No |
|  |  | [ ] Yes  [ ] No |
|  |  | [ ] Yes  [ ] No |

Notes: Feel free to add more rows as needed.

^a^ We recommend prioritizing factors your team believes may have the greatest impact on the program/practice’s implementation and are modifiable (via implementation strategies or other feasible changes in the organization or community).

***Brainstorming questions to help identify factors:***

- How might community member characteristics relate to use of the program/practice?
- How might community member health problems and needs relate to use of the program/practice?
- How might culturally relevant factors about the community members influence use of the program/practice?
- Which community member factors are most likely to influence program/practice impacts on health equity?
- Which community member factors does the team want to prioritize (e.g., assess or address in the future)?

**
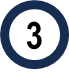

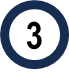
Organizational Factors Domain:**

**Brainstorming Template**

**Domain description**: Characteristics of the **A)** organizational context where the program or practice is implemented and delivered and **B)** organizational personnel. The organizations operate within a larger system and serve the community identified domain 2.

- 1. **Organization context factors:**

| Factor (name, brief description or definition) | How factor relates to health equity (positive or negative) | Prioritize? ^a^ |
| --- | --- | --- |
|  |  | [ ] Yes  [ ] No |
|  |  | [ ] Yes  [ ] No |
|  |  | [ ] Yes  [ ] No |
|  |  | [ ] Yes  [ ] No |
|  |  | [ ] Yes  [ ] No |

Notes: Feel free to add more rows as needed.

^a^ We recommend prioritizing factors your team believes may have the greatest impact on the program/practice’s implementation and are modifiable (via implementation strategies or other feasible changes in the organization or community).

***Brainstorming questions to help identify factors:***

- How could the organization’s policies and procedures influence use of the program/practice?
- How could the organization’s work culture and history influence use of the program/practice?
- How could the organization’s process for assigning work to staff influence the program/practice?
- Which organizational factors are most likely to influence the program/practice’s impacts on health equity?
- Which organizational factor(s) does the team want to prioritize (e.g., assess or address in the future)?
  1. **Organizational personnel factors:**

| Factor (name, brief description or definition) | How factor relates to health equity (positive or negative) | Prioritize? ^a^ |
| --- | --- | --- |
|  |  | [ ] Yes  [ ] No |
|  |  | [ ] Yes  [ ] No |
|  |  | [ ] Yes  [ ] No |
|  |  | [ ] Yes  [ ] No |
|  |  | [ ] Yes  [ ] No |

Notes: Feel free to add more rows as needed.

^a^ We recommend prioritizing factors your team believes may have the greatest impact on the program/practice’s implementation and are modifiable (via implementation strategies or other feasible changes in the organization or community).

***Brainstorming questions to help identify factors:***

- How could characteristics of staff (may include volunteers), supervisors or managers, and organizational leadership influence use of the program/practice?
- What culturally relevant factors about the staff, managers, and leadership might influence implementation of the program/practice?
- Which personnel factors are most likely to influence the program/practice’s impacts on health equity?
- Which personnel factor(s) does the team want to prioritize (e.g., assess or address in the future)?

**
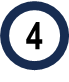

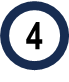
Community-Organization Interactions Domain:**

**Brainstorming Template**

**Domain description**: This domain focuses on interactions between community members (from domain 2) and organizational personnel (from domain 3) that take place as the program or practice is delivered. How these groups interact is very important from an equity perspective.

**Community Member-Organization Personnel Interactions:**

| Factor (name, brief description or definition) | How factor relates to health equity (positive or negative) | Prioritize? ^a^ |
| --- | --- | --- |
|  |  | [ ] Yes  [ ] No |
|  |  | [ ] Yes  [ ] No |
|  |  | [ ] Yes  [ ] No |
|  |  | [ ] Yes  [ ] No |
|  |  | [ ] Yes  [ ] No |

Notes: Feel free to add more rows as needed.

^a^ We recommend prioritizing factors your team believes may have the greatest impact on the program/practice’s implementation and are modifiable (via implementation strategies or other feasible changes in the organization or community).

***Brainstorming questions to help identify factors:***

- What modes of communication do community members and organizational personnel use? Do their preferences for communication mode and approach match?
- What is the level of trust and rapport between community members and organizational personnel? What has contributed to this and how might it influence their interactions?
- How do organizational personnel’s characteristics, priorities, etc. align with the needs and characteristics of community members?
- Which community-organization interaction factors are most likely to influence the program/practice’s impacts on health equity?
- Which community-organization interaction factor(s) does the team want to prioritize (e.g., assess or address in the future)?

**
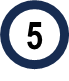

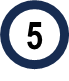
Bridging Factors Domain:**

**Brainstorming Template**

**Domain description**: Characteristics of the supports (such as workers, resources, and other infrastructure) available for implementing and sustaining the program/practice. This includes supports **A)** in the broader community, **B)** at the organizations, and **C)** supports that involve connections between the organizations and broader community (e.g., partnerships).

- - - 1. **Supports in the broader community:**

| Factor (name, brief description or definition) | How factor relates to health equity (positive or negative) | Prioritize? ^a^ |
| --- | --- | --- |
|  |  | [ ] Yes  [ ] No |
|  |  | [ ] Yes  [ ] No |
|  |  | [ ] Yes  [ ] No |
|  |  | [ ] Yes  [ ] No |
|  |  | [ ] Yes  [ ] No |

Notes: Feel free to add more rows as needed.

^a^ We recommend prioritizing factors your team believes may have the greatest impact on the program/practice’s implementation and are modifiable (via implementation strategies or other feasible changes in the organization or community).

***Brainstorming questions to help identify factors:***

- What types of external funding or in-kind support are currently available that might support implementation of the program/practice?
- What resources in the community context are currently available that might support implementation of the program/practice?
- Which community bridging factors are most likely to influence the program/practice’s impacts on health equity?
- Which community bridging factor(s) does the team want to prioritize (e.g., assess or address in the future)?
  - - 1. **Supports at the organizations:**

| Factor (name, brief description or definition) | How factor relates to health equity (positive or negative) | Prioritize? ^a^ |
| --- | --- | --- |
|  |  | [ ] Yes  [ ] No |
|  |  | [ ] Yes  [ ] No |
|  |  | [ ] Yes  [ ] No |
|  |  | [ ] Yes  [ ] No |
|  |  | [ ] Yes  [ ] No |

Notes: Feel free to add more rows as needed.

^a^ We recommend prioritizing factors your team believes may have the greatest impact on the program/practice’s implementation and are modifiable (via implementation strategies or other feasible changes in the organization or community).

***Brainstorming questions to help identify factors:***

- What resources within the organization could be available to support implementation of the program/practice?
- What quality assurance and quality improvement processes do the organizations currently use that could support implementation of the program/practice?
- Which organizational bridging factors are most likely to influence the program/practice’s impacts on health equity?
- Which organizational bridging factor(s) does the team want to prioritize (e.g., assess or address in the future)?
  - - 1. **Supports involving connections between the organizations and broader community:**

| Factor (name, brief description or definition) | How factor relates to health equity (positive or negative) | Prioritize? ^a^ |
| --- | --- | --- |
|  |  | [ ] Yes  [ ] No |
|  |  | [ ] Yes  [ ] No |
|  |  | [ ] Yes  [ ] No |
|  |  | [ ] Yes  [ ] No |
|  |  | [ ] Yes  [ ] No |

Notes: Feel free to add more rows as needed.

^a^ We recommend prioritizing factors your team believes may have the greatest impact on the program/practice’s implementation and are modifiable (via implementation strategies or other feasible changes in the organization or community).

***Brainstorming questions to help identify factors:***

- What is the strength and history of partnerships between the community and organizations, and how might this influence implementation of the program/practice?
- How could both organizational and community experts (such as trainers, consultants, coaches, etc.) support implementation of the program/practice?
- Which of these factors are most likely to influence the program/practice’s impacts on health equity?
- Which of these factor(s) does the team want to prioritize (e.g., assess or address in the future)?

**
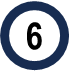

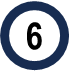
Process Factors Domain:**

**Brainstorming Template**

**Domain description**: Characteristics of how the organizations approach the process of implementing and sustaining the program/practice. Both who is involved and what they do together can have major impacts on the success of an implementation effort.

**Process factors:**

| Factor (name, brief description or definition) | How factor relates to health equity (positive or negative) | Prioritize? ^a^ |
| --- | --- | --- |
|  |  | [ ] Yes  [ ] No |
|  |  | [ ] Yes  [ ] No |
|  |  | [ ] Yes  [ ] No |
|  |  | [ ] Yes  [ ] No |
|  |  | [ ] Yes  [ ] No |

Notes: Feel free to add more rows as needed.

^a^ We recommend prioritizing factors your team believes may have the greatest impact on the program/practice’s implementation and are modifiable (via implementation strategies or other feasible changes in the organization or community).

**Brainstorming questions to help identify factors:**

- Who/what might be involved in engaging relevant people or groups in making changes?
- Who/what might be involved in assessing needs and context for the program/practice?
- Who/what might be involved in planning and executing changes for implementation of the program/practice?
- Who/what might be involved in evaluating the impact and outcomes of implementation?
- Who/what might be involved in identifying and making adaptations to improve the program/practice?
- Which process factors are most likely to influence the program/practice’s impacts on health equity?
- Which process factor(s) does the team want to prioritize (e.g., assess or address in the future)?


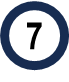

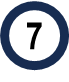
**Societal Context Domain:**

**Brainstorming Template**

**Domain description**: Characteristics of the broader society in which all the other domains exist. Factors from the societal context may not have unique effects on the program/practice but they can still have important, direct impacts on community needs and responses.

**Societal Context:**

| Factor (name, brief description or definition) | How factor relates to health equity (positive or negative) | Prioritize? ^a^ |
| --- | --- | --- |
|  |  | [ ] Yes  [ ] No |
|  |  | [ ] Yes  [ ] No |
|  |  | [ ] Yes  [ ] No |
|  |  | [ ] Yes  [ ] No |
|  |  | [ ] Yes  [ ] No |

Notes: Feel free to add more rows as needed.

^a^ We recommend prioritizing factors your team believes may have the greatest impact on the program/practice’s implementation and are modifiable (via implementation strategies or other feasible changes in the organization or community).

***Brainstorming questions to help identify factors:***

- How could economic conditions (related to work, affordable housing and food, etc.) influence implementation of the program/practice?
- How could environmental conditions (e.g., related to social or built environment such as economic, transportation, and housing conditions; natural environment such as air/water/food quality) influence implementation of the program/practice?
- How could sociopolitical forces (e.g., related to political parties and elections, social movements) influence implementation of the program/practice? This includes systems of oppression such as structural racism.
- Which societal factors are most likely to influence the program/practice’s impacts on health equity?
- Which societal factor(s) does the team want to prioritize (e.g., assess or address in the future)?

**CONGRATULATIONS!**

**You’ve reached the end of the brainstorming outlines.**

**Don’t forget to add the factors you’ve identified to the IM4Equity Template. This will provide an organized visual summary of the information discussed and prioritized.**

Remember, you can always contact us at [adopp@rand.org](mailto:adopp@rand.org) with any questions about using IM4Equity. We will be happy to help support your use of this tool.
